# Aptamer-Based Heterobifunctional Targeted Degraders in Disease Treatment

**DOI:** 10.3390/molecules31183318

**Published:** 2026-09-18

**Authors:** Xiuhua Liang, Menghan Du, Jing Wang, Xiaoyi Lei, Yinghong Tian, Xingmei Zhang

**Affiliations:** 1 Key Laboratory of Mental Health of the Ministry of Education, Guangdong-Hong Kong-Macao Greater Bay Area Center for Brain Science and Brain-Inspired Intelligence, Guangdong-Hong Kong Joint Laboratory for Psychiatric Disorders, Guangdong Province Key Laboratory of Psychiatric Disorders, Guangdong Basic Research Center of Excellence for Integrated Traditional and Western Medicine for Qingzhi Diseases, Department of Neurobiology, School of Basic Medical Sciences, Southern Medical University, Guangzhou 510515, Chinadmh200011@outlook.com (M.D.); wangjing20210226@163.com (J.W.); 2 First School of Clinical Medicine, The First Affiliated Hospital, Southern Medical University, Guangzhou 510515, China; lei0808xy@163.com; 3 Experiment Teaching & Administration Center, School of Basic Medical Sciences, Southern Medical University, Guangzhou 510515, China; tianyh@smu.edu.cn

**Keywords:** aptamer, heterobifunctional targeted degrader, PROTAC, LYTAC, ATTEC, disease, therapy

## Abstract

Aptamers are short nucleic acid molecules that bind to targets with high specificity. Recognized as “chemical antibodies”, they offer distinct advantages including strong programmability and ease of synthesis and chemical modification. Heterobifunctional targeted degraders were developed to address the challenges posed by traditionally “undruggable” targets and acquired drug resistance. The degraders recruit endogenous cellular degradation machinery to catalyze the removal of disease-associated proteins, thereby overcoming the limitations of conventional occupancy-driven inhibition. The fusion of aptamers with these degradation modalities has given rise to integrated platforms such as aptamer–proteolysis-targeting chimeras (PROTACs), aptamer–lysosome-targeting chimeras (LYTACs), and aptamer–autophagosome-tethering compounds (ATTECs). These conjugates combine the precise targeting ability of aptamers with the event-driven, catalytic nature of degradation technologies. As such, they not only expand the druggable target space to include intracellular proteins, membrane proteins, and pathogenic aggregates but also hold significant therapeutic potential in areas ranging from oncology and neurodegenerative disorders to infections and inflammatory diseases. Furthermore, degradation can be spatially and temporally controlled through receptor-mediated delivery or photo-switchable mechanisms. In this review, we provide a comprehensive summary of recent progress in studies on aptamer-based heterobifunctional degraders, with an emphasis on their design principles, biomedical applications, and therapeutic prospects.

## 1. Introduction

Since the initial development of aptamers and their screening techniques in 1990, namely, the Systematic Evolution of Ligands by Exponential Enrichment (SELEX) [1], the field has gradually garnered widespread attention. Aptamers are short, single-stranded DNA or RNA molecules selected using SELEX technology, which involve many rounds of binding screening and PCR amplification from oligonucleotide libraries with a large number of random sequences [2]. These molecules can fold into secondary structures, including stems, rings, loops, pseudoknots, G-quadruplexes, and kissing hairpins, via intramolecular base pairing [3]. These secondary structures together form a unique three-dimensional structure that can recognize and bind to various targets with high affinity and specificity [4]. Targets can include proteins, small molecules, metal ions, viral particles, bacteria, and even whole cells [5]. Because their binding mechanism resembles that in antibody–antigen interactions and they are amenable to large-scale production via solid-phase synthesis, aptamers are often called “chemical antibodies” [6]. They have shown great potential in molecular recognition and targeted interventions. Compared with antibodies, aptamers have lower molecular weights, are easily synthesized, and exhibit low immunogenicity [6], making them ideal substitutes for antibodies in targeted therapies [7], drug delivery [8], biomaterial development [9], and biosensing [10].

Aptamers have demonstrated considerable potential for diverse applications. Their high specificity and programmability enable the recognition of disease-specific biomarkers, such as the detection of low-abundance proteins in circulating tumor cells or exosomes in liquid biopsies, thereby supporting early diagnosis and personalized treatment strategies [11,12]. As therapeutic agents, aptamers can act as neutralizers or blockers by inhibiting enzyme activity or disrupting receptor–ligand interactions. For instance, the anti-VEGF aptamer Pegaptanib has been approved for clinical use in the management of wet age-related macular degeneration [13]. The CoV2-6C3 aptamer interferes with the interaction between the SARS-CoV-2 spike protein or its receptor-binding domain and the ACE2 receptor on host cells, effectively preventing viral infection [14]. Additionally, aptamers such as A1 [15], BI-1, and BI-2 [16] inhibit Beta-secretase 1 to ameliorate symptoms of Alzheimer’s disease. Aptamers can also serve as agonists to activate specific signaling pathways, exemplified by insulin receptor agonist aptamers [17]. Furthermore, aptamers may function as targeted carriers when either covalently or non-covalently conjugated with chemical drugs, nucleic acid therapeutics (such as siRNA or miRNA), peptides, proteins, or antibodies, to achieve selective drug accumulation at lesion sites, enhance therapeutic efficacy, and reduce systemic toxicity. For example, the ligand AS1411, which targets nucleolin (NCL), has been extensively evaluated to enhance the delivery efficiency of anticancer agents in tumor cells [18].

Traditional drug development has long relied on designing inhibitors or agonists targeting protein active sites to modulate target protein function [19]; however, it faces two core challenges. First, traditional methods struggle to target many disease-related proteins lacking well-defined active sites, such as transcription factor MYC [20] and KRAS [21]. Second, long-term use of single-target drugs readily induces resistance mutations, limiting their sustained efficacy [22]. To overcome these bottlenecks, targeted protein degradation (TPD) has emerged as a promising therapeutic strategy. These degraders no longer function as inhibitors; instead, they take advantage of the cell’s own protein degradation pathways, such as the ubiquitin-proteasome system (UPS) and the lysosomal pathway. By doing so, they enable the thorough removal of pathogenic proteins [23]. This “event-driven” catalytic approach not only holds promise for conquering “undruggable” targets but also offers advantages such as sustained activity, low dosage requirements, and high selectivity [24], and reduced potential toxicity [25]. Molecular glues [26] and proteolysis-targeting chimeras (PROTACs) [27] are two cornerstone modalities in the field of TPD, both of which leverage the UPS. Unlike bifunctional PROTACs, molecular glues are typically small, monovalent molecules that lack a chemical linker. They function by binding to E3 ubiquitin ligases to interact with target proteins, thereby promoting ubiquitination and subsequent proteasomal degradation. As representative heterobifunctional targeted degraders, PROTACs mainly target intracellular proteins. To further expand the scope of TPD, lysosome-targeting chimeras (LYTACs) [28] have also emerged, utilizing the lysosomal pathway to target extracellular and membrane proteins. Additionally, Autophagy-targeting chimeras and autophagosome-tethering compounds (ATTECs) degrade macromolecular aggregates or organelles via the autophagy pathway [29]. To date, multiple PROTACs have demonstrated significant therapeutic potential in oncology [30], neurodegenerative diseases [31], and autoimmune disorders [32].

Heterobifunctional targeted degraders that rely on traditional small-molecule ligands face considerable practical limitations. For example, the advancement of PROTACs is hampered by the challenge of identifying suitable ligands for the proteins of interest (POI) and the E3 ubiquitin ligase. From an application standpoint, many small-molecule PROTACs exhibit limited solubility, suboptimal cellular permeability, and unfavorable pharmacokinetic properties because of their relatively large size and high polar surface area [33]. These physicochemical limitations can compromise intracellular exposure and may contribute to off-target effects and nonspecific toxicity. Additionally, PROTAC activity is governed by the formation of a productive ternary complex rather than by binary target engagement alone. Consequently, the relationship between degrader concentration and degradation efficacy is frequently non-monotonic. At excessive concentrations, PROTAC molecules can preferentially occupy the target protein or E3 ligase as non-productive binary complexes, thereby competing with productive target–PROTAC–E3 ternary complex formation and reducing degradation efficacy, a phenomenon known as the “hook effect” or prozone effect [34]. This effect is closely associated with ternary-complex cooperativity, avidity, and the relative kinetics of complex formation and dissociation. Positive cooperativity and prolonged ternary-complex residence can broaden the concentration window for productive degradation, whereas weak or negatively cooperative ternary interactions can make the hook effect more pronounced. Thus, optimization of PROTAC efficacy requires consideration not only of binary binding affinity but also of ternary-complex geometry, cooperativity, and kinetic stability. Systemic administration also raises concerns about potential off-target toxicity in healthy tissues expressing the chosen E3 ligase [35], which may hinder clinical translation.

Aptamers are structurally dynamic nucleic acid ligands whose target recognition can involve extensive surface contacts and conformational rearrangement, and their binding behavior is determined by both association and dissociation kinetics. Recent kinetic analyses of aptamer–target interactions have demonstrated substantial variation in k_on_ and k_off_ among different aptamers [36], indicating that binding kinetics may differ considerably from those of conventional small-molecule ligands. In principle, these features may alter the formation, stability, and residence time of aptamer-mediated ternary complexes and consequently influence the concentration range over which productive degradation occurs. However, direct quantitative comparisons of hook effects and ternary-complex kinetics between aptamer-based degraders and conventional small-molecule PROTACs remain limited. Therefore, the hook effect should be regarded as a general mechanistic consideration for aptamer-based heterobifunctional degraders rather than a phenomenon that is necessarily eliminated by the larger size of aptamers. Systematic characterization of concentration-dependent degradation profiles, ternary-complex cooperativity, and k_on_/k_off_ parameters will be important for determining whether the distinctive structural and kinetic properties of aptamers provide a broader or otherwise altered degradation window.

Aptamer-based strategies offer a promising alternative for expanding the scope of heterobifunctional targeted degraders, including to membrane and extracellular proteins. Compared with antibodies, aptamers are substantially smaller than antibodies, which makes them readily amenable to chemical modification and scalable production. They generally exhibit low immunogenicity and offer considerable flexibility for molecular engineering and targeted delivery [37]. In selected contexts, they may also provide favorable tissue penetration and receptor-mediated internalization; however, efficient cytosolic delivery remains a substantial hurdle. It should be acknowledged that cellular internalization is not an intrinsic property of all aptamers but is influenced by aptamer sequence and structural features, the identity and endocytic properties of the target receptor, as well as chemical modifications and delivery formats [38,39,40,41]. As opposed to small-molecule ligands, aptamers do not depend on binding to defined active site pockets, enabling them to engage a broader range of protein classes, including traditionally “undruggable” targets such as RNA-binding proteins, transcription factors, and G-quadruplex-binding proteins [5]. Aptamers, therefore, provide a versatile recognition platform for addressing targets that are challenging due to ligand scarcity or subcellular localization constraints. Recent advances in PROTAC design have further highlighted the importance of systematically optimizing the protein-of-interest ligand, E3 ligase ligand, and linker, with nucleic acid ligands such as aptamers providing an alternative strategy for engaging challenging targets [42].

Several reviews have previously summarized the emerging field of aptamer-based TPD. Tan et al. provided an early perspective on aptamer-based TPD, outlining the rationale for integrating aptamers with degradation technologies and discussing representative applications and translational challenges [43]. In parallel, a review published by Xie et al. highlighted the potential of aptamers to improve degrader delivery and enable spatiotemporal control of protein degradation [44]. The subsequent review by Li et al. further summarized advances in aptamer-based TPD, particularly aptamer–PROTAC and aptamer–LYTAC systems, with an emphasis on their therapeutic potential and challenges for clinical translation [44]. These studies have established an important conceptual and experimental foundation for the field. However, the rapid expansion of aptamer-based degradation technologies, including the emergence of aptamer–ATTECs, covalent and photoresponsive degraders, multivalent and multifunctional architectures, and applications beyond oncology, has substantially broadened the scope of this field. Therefore, an updated framework that systematically distinguishes these architectures while integrating their molecular design principles, degradation mechanisms, disease applications, and translational barriers is warranted. In this review, we provide such an updated perspective by comprehensively examining aptamer-based heterobifunctional targeted degraders, with particular emphasis on aptamer–PROTACs, aptamer–LYTACs, and the emerging aptamer–ATTEC platform.

## 2. Disease Treatment

### 2.1. Aptamer–PROTACs

PROTACs are emerging therapeutic agents that degrade the POI by recruiting E3 ubiquitin ligases. These molecules facilitate the spatial proximity of the POI and an E3 ligase, resulting in the formation of a ternary complex that promotes ubiquitination and subsequent degradation via the UPS. A typical PROTAC consists of three elements: a ligand that binds the POI (e.g., a kinase or nuclear receptor), a second ligand that recruits a specific E3 ligase, such as CRBN or VHL, and a linker that optimizes spatial orientation to facilitate ternary complex formation [19]. In 2001, Sakamoto et al. developed the first generation of degraders by combining target protein ligands with E3 ligase ligands, achieving successful degradation of methionine aminopeptidase-2 (MetAP-2) [27]. Since MetAP-2 degradation inhibits angiogenesis, this strategy offered a potential means to suppress tumor growth and metastasis. In contrast to conventional inhibitors, which typically require sustained exposure at high concentrations to maintain efficacy, PROTACs function via a catalytic mechanism that markedly lowers the required therapeutic dose, thereby reducing dose-dependent toxicity and the risk of drug resistance [45].

#### 2.1.1. Aptamer-Mediated Delivery of Conventional PROTACs

**Figure 1:**
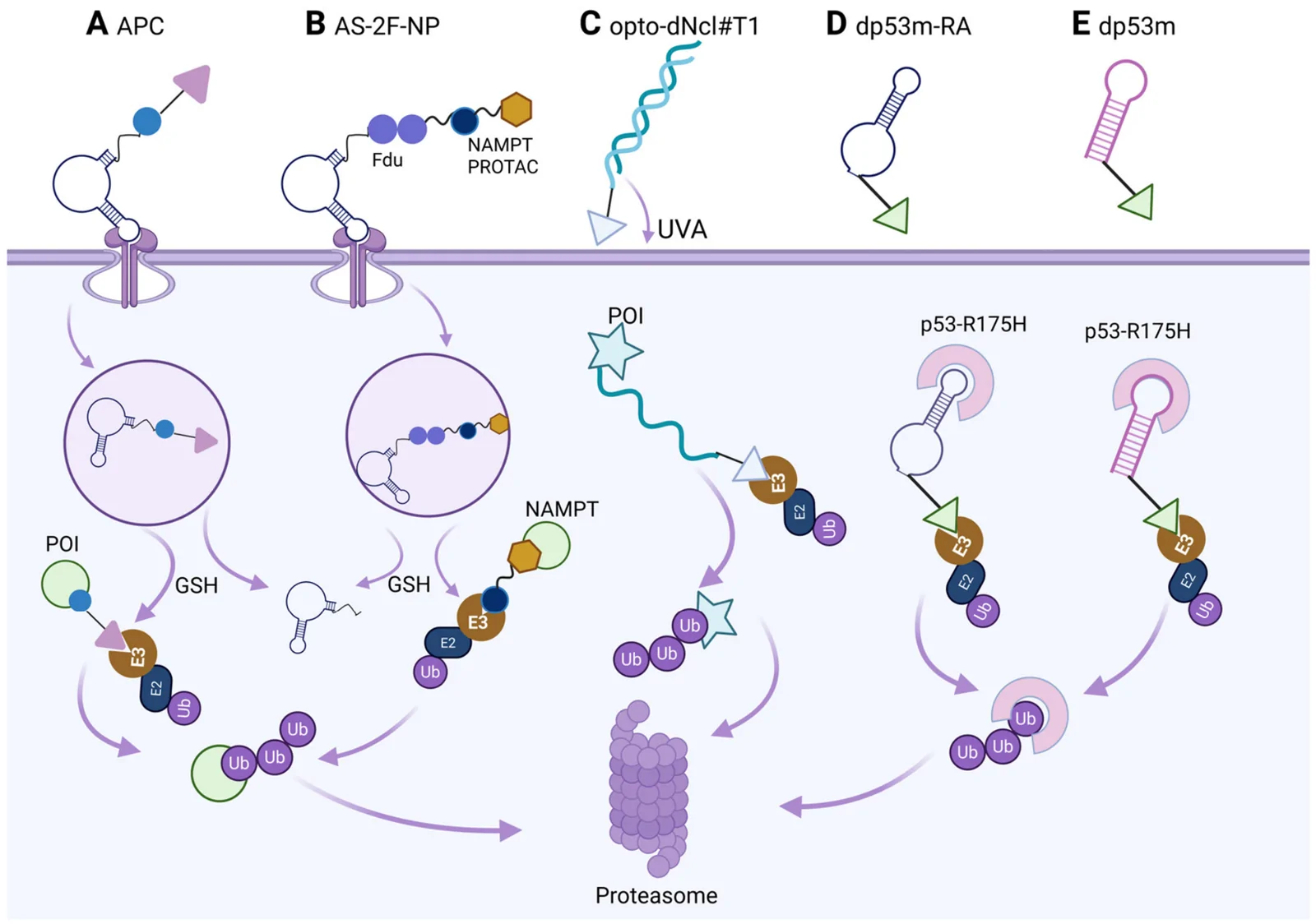
Schematic of aptamer-based proteolysis-targeting chimeras (PROTACs). Arrows indicate the proposed mechanistic sequence, including target recognition and, where applicable, cellular internalization, formation of degrader–target–effector complexes, recruitment of the ubiquitination machinery, target ubiquitination, and subsequent proteasomal degradation. (**A**) The first aptamer-PROTAC conjugate (APC) was constructed by linking a BET-targeting PROTAC to the nucleolin-targeting aptamer AS1411 (AS) through a cleavable disulfide bridge. Following cellular uptake, elevated intracellular glutathione (GSH) cleaves the disulfide bond, resulting in the release of the PROTAC, which induces degradation of protein of interest (POI) via the proteasome pathway. (**B**) The conjugate is constructed by tethering AS1411 to a nicotinamide phosphoribosyltransferase (NAMPT)-directed PROTAC (NP) via a GSH-responsive cleavable linker, with two fluorouridine (Fdu) nucleotides integrated into the aptamer sequence. Upon tumor cell internalization mediated by aptamer recognition, the GSH-sensitive linker is cleaved intracellularly to release the active NP. (**C**) Opto-apt-PROTAC is assembled by hybridizing a photo-labile complementary oligonucleotide to an aptamer-PROTAC. Upon UVA irradiation, the precursor opto-dNCL#T1 releases active dNCL#T1, which induces degradation of nucleolin (NCL) via the proteasome pathway. (**D**) Strategy for targeted degradation of p53-R175H using dp53m-RA. This conjugate comprises three elements: an E3 ligase ligand, a linker, and a p53m-RA aptamer specific for p53-R175H. By simultaneously engaging the target protein and the E3 ubiquitin ligase, dp53m-RA promotes ubiquitination and subsequent proteasomal degradation of p53-R175H. (**E**) Structure of dp53m, which is analogous to dp53m-RA except that the p53-R175H-targeting aptamer p53m-RA is replaced by p53m-DA.

Aptamers can serve as targeting ligands that recognize cell-surface receptors or other disease-associated biomarkers, promoting the selective internalization of PROTACs into target cells. This strategy has been exemplified by Sheng et al., who developed a novel aptamer–PROTAC conjugate (APC) by conjugating the NCL-targeting aptamer AS1411 with a BET-targeting PROTAC (Figure 1A). AS1411 was employed primarily as a delivery vehicle to transport PROTAC molecules into breast cancer cells, enhancing tumor-specific targeting while minimizing off-target effects in normal tissues. Compared to treatment with PROTAC alone, the APC exhibited improved in vivo degradation of BET proteins, enhanced antitumor efficacy in MCF-7 xenograft models, and reduced systemic toxicity [46].

A common limitation of aptamer-drug conjugates is their constrained drug-loading capacity. The sequential conjugation of multiple drug molecules to a single aptamer also remains challenging. To overcome this issue, Sheng et al. subsequently developed a related multifunctional smart prodrug system termed a “drugtamer-PROTAC conjugate,” which incorporates the aptamer AS1411, the cytotoxic agent Fdu, and a nicotinamide phosphoribosyltransferase (NAMPT) PROTAC (Figure 1B). Leveraging the structural similarities between Fdu, and thymidine nucleotides, Fdu was directly incorporated into the oligonucleotide chain during solid-phase aptamer synthesis. This strategy enabled the precise co-delivery of Fdu and the NAMPT–PROTAC within a single molecule. The resulting conjugate, AS-2F-NP, displayed enhanced aqueous solubility and improved cellular uptake, leading to enhanced degradation of the POI at the cellular level [47]. This system allowed for simultaneous tumor-specific targeting, efficient intracellular delivery, and synergistic combination therapy.

#### 2.1.2. Aptamer-PROTACs Using Aptamers as POI-Binding Ligands

##### Aptamer–PROTACs for Targeted Cancer Treatment

A conceptually distinct approach is to use the aptamer directly as the POI-recognition module. Here, the aptamer replaces the traditional small-molecule ligand by binding the target, while an E3 ligase recruiter is conjugated to it through a suitable linker. This design yields a bona fide aptamer-based degrader, as opposed to an aptamer-mediated delivery system that merely shuttles a preformed conventional PROTAC. Xie et al. provided a representative example. Using the NCL-specific aptamer AS1411, the first APC targeting NCL, designated dNCL#0, was synthesized by conjugating the aptamer to a CRBN E3 ligase ligand (CRBNL) via click chemistry. Through systematic linker optimization, the researchers identified a superior derivative, dNCL#T1, which effectively triggered the degradation of NCL via a ubiquitin-dependent pathway. This molecule significantly suppressed cell proliferation, migration, and tumor growth across multiple breast cancer cell models. To address potential toxicity associated with continuous PROTAC activity, the team further engineered a photoactivatable APC, termed opto-dNCL#T1 (Figure 1C). This construct was designed with a complementary photosensitive oligonucleotide strand that kept the molecule in an inactive “locked” state in light-free conditions. Upon UVA irradiation, the caging strand cleaved, releasing active dNCL#T1 and enabling the spatiotemporally controlled degradation of NCL [48]. This strategy not only improved biosafety but also provided a generalizable method for achieving precisely regulating APC activity.

The tumor suppressor p53, encoded by the most frequently mutated gene TP53 in human cancers, is often dysfunctional in various malignancies [49]. Among its mutants, p53-R175H represents a common hotspot variant strongly linked to tumor initiation, metastatic spread, heightened drug resistance, and unfavorable clinical outcomes [50]. To address this challenging target, Xie et al. developed p53m-RA, an RNA aptamer that selectively binds p53-R175H. Building on this recognition element, the first PROTAC molecule specifically targeting this oncogenic mutant was designed. Using click chemistry, an alkynylated CRBNL was conjugated to the 5′-azide-modified p53m-RA aptamer, generating a selective degrader termed dp53m-RA [51]. dp53m-RA (Figure 1D) induced the dose-dependent degradation of the mutant protein while selectively preserving wild-type (WT) p53, suggesting that dp53m-RA holds therapeutic promise for cancers harboring the p53-R175H mutation.

Previous studies demonstrated that the RNA aptamer p53m-RA binds the p53-R175H mutant with approximately tenfold higher affinity than to p53-WT, yet its poor serum stability hindered practical application. To overcome this limitation, Xie applied molecular dynamics simulations to guide an iterative Post-SELEX optimization strategy, systematically modifying non-core nucleotides and engineering a DNA-based version termed p53m-DA. The resulting DNA aptamer not only retained high specificity for p53-R175H, but also exhibited markedly improved stability in sera. To generate a PROTAC, p53m-DA was conjugated to a CRBNL via click chemistry, yielding the degrader molecule dp53m (Figure 1E). dp53m induced the degradation of the p53-R175H protein, effectively inhibiting the proliferation and migration of p53-R175H-driven cancer cells in vitro. Furthermore, in vivo dp53m treatment significantly suppresses tumor growth. Combining dp53m with cisplatin synergistically suppressed proliferation of p53-R175H-expressing cancer cells [52]. The potential of dp53m, as monotherapy or in conjunction with standard chemotherapeutic regimens, to treat tumors harboring the p53-R175H mutation is therefore conceivable.

**Figure 2:**
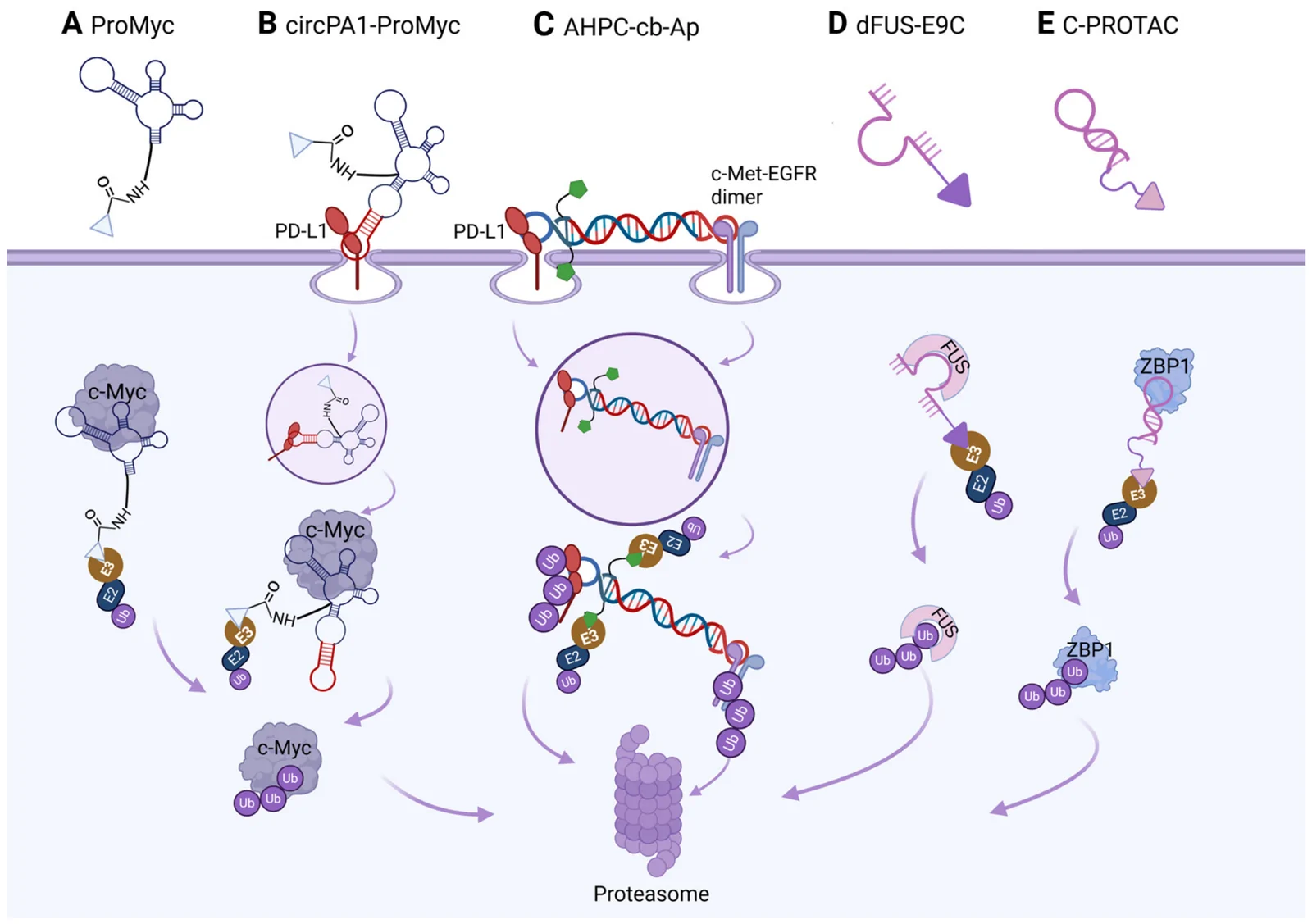
Schematic of aptamer-based proteolysis-targeting chimeras (PROTACs). Arrows indicate the proposed mechanistic sequence, including target recognition and, where applicable, cellular internalization, formation of degrader–target–effector complexes, recruitment of the ubiquitination machinery, target ubiquitination, and subsequent proteasomal degradation. (**A**) ProMyc is designed to degrade c-Myc via the proteasome. It comprises a c-Myc-specific aptamer and an E3 ligase ligand, which together mediate the ubiquitination and subsequent degradation of c-Myc. (**B**) circPA1-ProMyc is engineered to bind intracellular c-Myc. Compared with ProMyc, it incorporates artificial cyclization and an anti-PD-L1 aptamer to improve stability and delivery efficiency. (**C**) AHPC-cb-Ap is constructed by ligating the c-Met-targeting aptamer SL1 and the PD-L1-targeting aptamer Mj5c into a circularized bivalent structure (cb-Ap), which is then conjugated with two molecules of the E3 ligase ligand (S,R,S)-AHPC. Due to the heterodimeric interaction between c-Met and other receptor tyrosine kinases (e.g., EGFR), their co-degradation is simultaneously triggered. (**D**) dFUS-E9C is a bifunctional RNA-CRBN conjugate that selectively binds FUS via its RGG2/RRM domains and recruits the E3 ligase. This ternary complex induces FUS ubiquitination and proteasomal degradation, effectively clearing cytoplasmic FUS aggregates and pathogenic mutants without altering FUS mRNA levels. (**E**) C-PROTAC targets Z-DNA-binding protein 1 (ZBP1) for degradation and contains three components: an E3 ligase ligand, a linker, and a ZBP1-specific aptamer. This conjugate facilitates ZBP1 ubiquitination and proteasomal degradation.

The transcription factor c-Myc functions as a potent oncoprotein and has been implicated in approximately 70% of human cancers, wherein it drives tumorigenesis and confers treatment resistance [53]. Its intrinsic structural disorder, absence of well-defined active sites, nuclear localization, and potential for on-target toxicity in normal tissues have collectively rendered c-Myc “undruggable” by conventional small-molecule or antibody-based approaches [54]. To overcome these limitations, Zhao developed a high-throughput screening platform termed microwell-SELEX, which employs polystyrene microarrays to efficiently select aptamers against unlabeled proteins. Using this system, a DNA aptamer with high specificity for c-Myc was identified. Building on this recognition element, ProMyc, a multifunctional degrader, was engineered to not only deplete the c-Myc protein via a heterobifunctional mechanism but also downregulate its essential binding partner Max, resulting in synergistic suppression of c-Myc’s transcriptional activity (Figure 2A. To further improve biostability and delivery, PA1—an artificially cyclizable aptamer with anti–PD-L1 activity—was incorporated into a circularized complex designated circPA1-ProMyc [55]. In xenograft tumor models, this construct significantly inhibited tumorigenesis, highlighting its potential as an innovative therapeutic option for addressing c-Myc–dependent malignancies (Figure 2B).

Non-small cell lung cancer (NSCLC) represents the most common histological category among lung cancer subtypes. A key oncogenic driver in NSCLC is mutation in the epidermal growth factor receptor (EGFR), making EGFR tyrosine kinase inhibitors (EGFR-TKIs) the standard first-line therapy for patients harboring such mutations [56]. However, long-term treatment often leads to acquired resistance, primarily mediated by secondary EGFR mutations or activation of bypass signaling pathways such as c-Met. To overcome this limitation, Tan devised a dual-function strategy to counteract EGFR-TKI resistance in NSCLC by developing a cyclized bivalent aptamer-based PROTAC, termed AHPC-cb-Ap (Figure 2C). This molecule enabled the simultaneous and efficient degradation of c-Met and multiple classes of tyrosine kinases that interact with it, including EGFR. In vitro and in vivo experiments demonstrated that AHPC-cb-Ap treatment restored sensitivity in NCI-H1975 cells that had developed resistance to erlotinib owing to the EGFR T790M/L858R mutation. Notably, AHPC-cb-Ap not only degraded c-Met and EGFR but also reduced the protein levels of other c-Met-interacting partners, such as Her2 and Her3, highlighting its broad-spectrum potential to counteract drug resistance [57]. This strategy provided a comprehensive and potent degradation-based approach to reverse NSCLC resistance and can be extended to other cancer types, offering a promising new tool for personalized therapies.

##### Aptamer–PROTACs for Targeted Neurodegeneration Treatment

Frontotemporal dementia and amyotrophic lateral sclerosis share a common pathological hallmark: the abnormal aggregation and mislocalization of the RNA-binding protein FUS, particularly the cytoplasmic accumulation of its mutant forms [58,59]. While conventional oligonucleotide therapies have made some advances, they remain limited by two major challenges: inefficient blood–brain barrier (BBB) penetration and the inability to rapidly clear toxic proteins, which restrict their therapeutic utility in central nervous system disorders. To overcome these limitations, Xie et al. developed a brain-penetrating DNA nanoflower platform. By integrating a FUS-targeting RNA oligonucleotide with CRBNL, the team constructed a novel oligo–PROTAC molecule termed dFUS-E9C (Figure 2D). This system incorporated transferrin receptor (TfR) aptamers on the nanoflower surface, enabling efficient BBB traversal via TfR-mediated transport to deliver the active molecules into the brain. Once inside, the UPS was recruited through CRBN to selectively degrade FUS and its disease-associated variants. dFUS-E9C efficiently degraded FUS with sustained degradation, exhibiting high specificity and significantly alleviating mitochondrial damage, elevated reactive oxygen species (ROS) levels, and cell death induced by FUS overexpression, while restoring mitochondrial membrane potential [60]. Using FUS as a proof-of-concept target, the degradation efficiency, specificity, and therapeutic potential of dFUS-E9C were systematically evaluated in both cellular and animal models. This technology offers a novel integrated strategy for treating neurodegenerative diseases by combining engineered oligonucleotides with targeted protein-degradation technologies.

##### Aptamer–PROTACs for Targeted Anti-Inflammatory Treatment

In addition to their applications in cancer therapy and neurodegenerative disorders, aptamers are being leveraged to develop antiviral and anti-inflammatory agents. Although inflammation triggered by viral infection is a vital host defense mechanism, its overactivation can result in tissue damage and disease progression [61,62]. Recent studies have shown that upon pathogenic stimulation, Z-DNA-binding protein 1 (ZBP1) recognizes Z-form nucleic acids (Z-DNA or Z-RNA) and activates cell death pathways, thereby inducing a potent inflammatory response [63,64]. To target this domain, Liu et al. used SELEX to screen a panel of nucleic acid aptamers against ZBP1. Using surface plasmon resonance, Z3 was identified as the highest-affinity candidate with a binding strength exceeding that of ZBP1’s natural interaction with Z-DNA. A covalent PROTAC (C-PROTAC) was developed using the Z3 aptamer. This molecule consisted of three modules: a ZBP1-specific DNA aptamer, a linker unit capable of forming a covalent bond with the target protein via an N-acyl-N-alkyl sulphonamide bridge, and a ligand that recruits E3 ubiquitin ligases (Figure 2E). Upon binding to ZBP1, C-PROTAC covalently stabilized the complex and directed the E3 ligase-mediated ubiquitination of ZBP1, resulting in its subsequent degradation via the proteasome [65]. This approach effectively inhibited ZBP1-driven necroptosis and mitigated excessive inflammation, representing a promising treatment approach for infection-associated inflammatory diseases and providing a basis for the development of next-generation antiviral and anti-inflammatory therapeutics. Nevertheless, the irreversible nature of covalent target engagement introduces additional safety considerations for therapeutic development. Unlike non-covalent degraders, which can dissociate from the target when systemic exposure decreases, irreversible covalent degraders may maintain target occupancy for prolonged periods and may not readily recover from unintended target engagement [66,67]. Moreover, insufficiently selective covalent reactivity could result in modification of off-target proteins, potentially causing prolonged or cumulative toxicity [68]. Therefore, the therapeutic window of C-PROTACs depends not only on aptamer-mediated target recognition but also on the chemical selectivity and reaction kinetics of the covalent linkage.

### 2.2. Aptamer–LYTACs

Unlike PROTACs, which primarily exploit the UPS to degrade intracellular proteins, extracellular and membrane-TPD (emTPD) employs distinct trafficking and degradation mechanisms to eliminate proteins that are inaccessible to conventional intracellular degraders. Recent advances have categorized emTPD strategies into receptor-hijacking, surface E3 ligase recruitment, and receptor-independent or programmable routing mechanisms, highlighting endolysosomal trafficking as a key determinant of degradation efficiency, tissue selectivity, and durability. LYTACs represent an important receptor-hijacking strategy within this broader framework, enabling the degradation of secreted and membrane-associated proteins through receptor-mediated internalization and lysosomal delivery [69]. The first-generation LYTAC, developed by the Bertozzi in 2020 [28], is a heterobifunctional molecule. It consists of two binding moieties: an oligoglycopeptide group and a ligand for the POI connected by a linker. The oligoglycopeptide is recognized by the cation-independent mannose-6-phosphate receptor (CI-M6PR) on the cell surface. This recognition triggers receptor-mediated endocytosis, internalizing the target protein–LYTAC–CI-M6PR complex within a vesicle. The vesicle sequentially matures into an early and then a late endosome, which subsequently fuse with a lysosome to form an endolysosome, where the target protein is ultimately degraded [28]. Operating through an event-driven mechanism similar to that of PROTACs, various LYTACs have been engineered to degrade proteins such as EGFR, PD-L1, apolipoprotein E4, and other pathogenic proteins implicated in Alzheimer’s disease and cancer [23,28]. Building on first-generation LYTACs, which lacked tissue specificity and carried potential toxicity risks, Bertozzi introduced an asialoglycoprotein receptor (ASGPR)-based LYTAC platform [70]. This leveraged ASGPR, a lysosomal shuttle receptor predominantly expressed in hepatocytes, enabling liver-specific degradation of cell surface targets [71]. These antibody-based LYTACs typically comprise an antibody linked to a lysosome-targeting ligand, such as mannose-6-phosphate (M6Pn) or N-acetylgalactosamine (GalNAc). However, their production complexity, structural heterogeneity, and suboptimal endocytic efficiency highlight the need for alternative, small-molecule-based systems.

#### 2.2.1. Aptamer–LYTAC for Targeted Cancer Treatment

**Figure 3:**
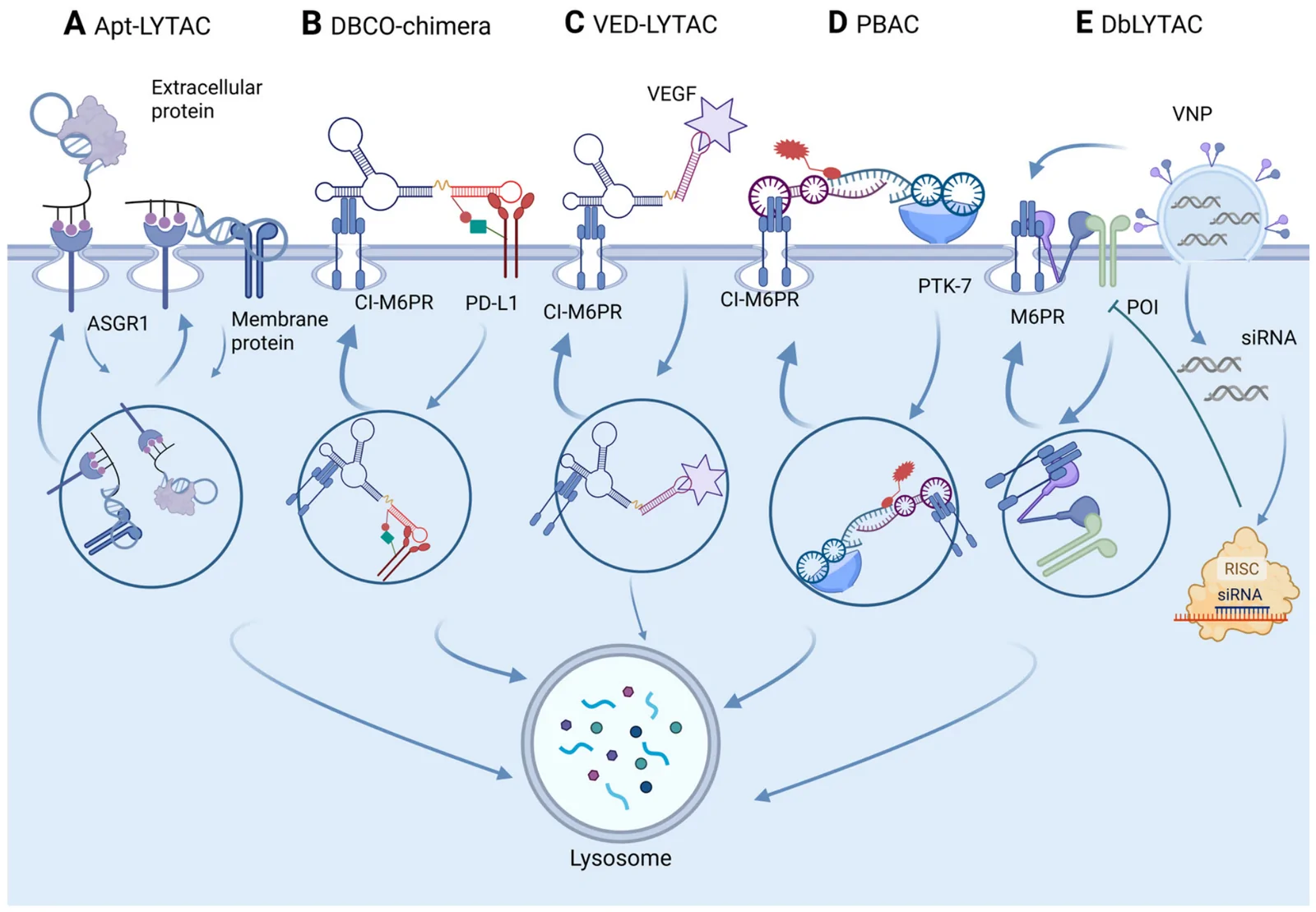
Schematic illustration of aptamer-based LYTACs. Arrows indicate the sequential events involved in lysosome-targeting degradation, including target recognition and binding, receptor-mediated internalization, intracellular trafficking to lysosomes, subsequent lysosomal degradation, and recycling of the internalized receptor back to the plasma membrane. (**A**) Apt-LYTAC is composed of an aptamer directed against the protein of interest (POI) and a trivalent N-acetylgalactosamine (tri-GalNAc) motif targeting asialoglycoprotein receptor (ASGPR). This dual-module structure facilitates liver-specific lysosomal degradation of extracellular and membrane-associated proteins. (**B**) DBCO-chimera is a bispecific aptamer construct linking a PD-L1-binding aptamer to a cation-independent mannose-6-phosphate receptor (CI-M6PR)-targeting aptamer via a chemical linker. Simultaneous engagement of both receptors redirects PD-L1 to lysosomes for degradation. (**C**) VED-LYTAC is a bispecific aptamer-based constructs linking a VEGF-binding aptamer to a CI-M6PR-targeting aptamer via a double-stranded DNA linker. Simultaneous engagement of both receptors redirects VEGF to lysosomes for degradation, thereby suppressing VEGF-induced angiogenesis and vascular leakage. (**D**) Similarly, PBAC incorporates a PTK7-targeting aptamer and a CI-M6PR-targeting aptamer connected by short oligonucleotide linkers. Notably, near-infrared (NIR) laser irradiation enhances the lysosomal degradation efficiency of this construct. (**E**) VNP combines LYTAC and gene silencing functions. It mediates membrane fusion to deliver siRNA, then forms RNA-induced silencing complex (RISC), while DbLYTAC is anchored on the cell surface to direct target proteins toward lysosomal degradation. DbLYTAC is constructed by two modules: a M6PR aptamer and a POI aptamer, which were linked by a click reaction.

To overcome these limitations, Zhu et al. developed an aptamer–LYTAC strategy. In this design, the tri-GalNAc moiety was conjugated to a nucleic acid aptamer. The aptamer could bind to extracellular or membrane-associated proteins, while tri-GalNAc specifically recruited ASGPR. Upon co-binding, the ternary complex was internalized via receptor-mediated endocytosis, while the target protein was trafficked to the lysosome for degradation. As a proof-of-concept, the team synthesized an aptamer–LYTAC by conjugating tri-GalNAc to an aptamer targeting platelet-derived growth factor BB (PDGF-BB), a driver of cancer cell proliferation, invasion, and metastasis (Figure 3A). In ASGPR-positive HepG2 cells, the GalNAc-Apt_PDGF_ construct significantly enhanced the internalization and lysosomal degradation of fluorescently labeled PDGF. To further explore the platform’s capability for degrading membrane proteins, the aptamer was replaced with Sgc8c, which specifically binds to the transmembrane receptor protein tyrosine kinase 7 (PTK7). PTK7 is overexpressed in tumors and regulates the Wnt signaling network, thereby influencing cell motility and invasion. The resulting GalNAc-Apt_PTK7_ reduced PTK7 levels on the cell surface in HepG2 cells in a time- and concentration-dependent manner [72]. Aptamer–LYTACs therefore represent a versatile strategy for achieving the cell type-specific degradation of extracellular and membrane proteins, offering a potential targeted therapeutic strategy for hepatocellular carcinoma and other diseases.

Following the introduction of the first-generation LYTAC technology, Bertozzi demonstrated its ability to degrade the PD-L1 protein. However, when such chimeric molecules rely solely on non-covalent interactions, such as hydrogen bonds, for protein targeting, their retention at the target site may be insufficient, potentially compromising degradation efficiency in complex in vivo environments. To address this limitation, Liu et al. introduced a covalent stabilization strategy to enhance the target residence of LYTACs and improve membrane protein degradation. The team designed a dual-functional chimeric aptamer, termed DBCO-chimera, which combined the precise targeting and specificity of aptamers with a bioorthogonal covalent linkage via the DBCO group (Figure 3B). The DBCO-chimera mediated PD-L1 depletion through a dual immunostimulatory mechanism; it simultaneously blocked the PD-1/PD-L1 immunosuppressive axis and induced immunogenic apoptosis in tumor cells. PD-L1 degradation was further associated with profound alterations in intracellular signaling, particularly the suppression of p-STAT3 dimerization, which can lead to the upregulation of pro-apoptotic proteins. Apoptotic cells released increased levels of damage-associated molecular patterns (DAMPs), including calreticulin, ATP, and high-mobility group box 1, upon treatment with the covalent LYTAC. These DAMPs effectively promoted dendritic cell maturation and antigen-presenting function and substantially enhanced T cell activation [73].

#### 2.2.2. Bispecific Aptamer–LYTAC for Targeted VEGF Degradation

A notable application of aptamer-based LYTACs beyond oncology was reported by Zhou et al., who developed a bispecific VEGF-degrading LYTAC (VED-LYTAC) for the treatment of pathological retinal angiogenesis. VED-LYTACs comprise a VEGF-binding aptamer, an M6PR-binding aptamer, and a DNA linker, thereby simultaneously engaging extracellular VEGF and M6PR (Figure 3C). This interaction promotes M6PR-mediated internalization of the VEGF–VED-LYTAC complex and subsequent lysosomal degradation of VEGF. Among the constructs evaluated, optimized VED-LYTACs effectively reduced VEGF levels and suppressed VEGF-driven angiogenic responses in vitro and ex vivo. Importantly, intravitreal administration of VED-LYTACs alleviated pathological retinal neovascularization and vascular leakage in mouse models of neovascular ocular disease. This study extends aptamer–LYTAC applications from membrane-associated targets to soluble extracellular growth factors and demonstrates the potential of targeted protein degradation as an alternative to conventional VEGF blockade in ocular vascular disorders [74].

#### 2.2.3. Light-Responsive Platform PT-LYTAC Enables Precise Structural Control

To enhance the structural precision and therapeutic performance of aptamer–LYTACs, Tan developed a photoactive bispecific aptamer chimera (PBAC) designed to improve the targeted degradation of membrane proteins. The construct was assembled by conjugating a near-infrared photosensitizer (PA) to the PTK7-targeting aptamer Sgc8 via copper-free click chemistry, yielding Sgc8-PA. This unit was then hybridized with an aptamer targeting the lysosomal shuttling receptor, CI-M6PR, via complementary DNA strand assembly, resulting in the final PBAC structure (Figure 3D). The team named this light-responsive platform PT-LYTAC. PT-LYTAC operates through a sequential mechanism: initially, PBAC enables bispecific recognition that precisely binds and delivers PTK7 to lysosomes. Subsequent irradiation with a 660 nm laser induces ROS generation, which activates the autophagy pathway. This process establishes a self-amplifying “light-triggered autophagy–degradation” feedback loop, thereby substantially enhancing the clearance of the target membrane protein and providing the multi-dimensional suppression of cancer cell proliferation and migration [75]. This platform showcases a modular design strategy that leverages the intrinsic programmability of DNA aptamers to enable precise structural control. It combines straightforward preparation, a low effective dose, and high degradation efficiency, thereby offering a versatile and innovative platform for membrane protein degradation therapeutics.

#### 2.2.4. Multimodal and Multitarget Aptamer-LYTAC Platforms

While antibody-based LYTAC platforms can promote lysosomal degradation of membrane proteins by engaging lysosomal trafficking receptors, their clinical application is often limited by their macromolecular complexity and intrinsic immunogenicity. To tackle these limitations, Zhang et al. developed a modular, multifunctional, and highly stable virus-like nanoparticle (VNP) system. Their design employs a DNA tetrahedron scaffold functionalized at one terminus with an aptamer targeting a membrane protein (e.g., MET or EGFR) and another an aptamer targeting the lysosomal receptor M6PR, creating a modular construct termed DbLYTAC (Figure 3E). Importantly, the two functional modules act at complementary levels of protein homeostasis rather than representing redundant mechanisms: siRNA mediates post-transcriptional silencing of target mRNA and thereby suppresses de novo protein synthesis, whereas DbLYTAC promotes lysosomal removal of pre-existing cell-surface protein. This temporal and mechanistic complementarity provides a rationale for combining the two modalities, because depletion of existing protein by LYTAC does not directly prevent subsequent protein resynthesis, while siRNA-mediated mRNA suppression alone does not rapidly eliminate the pre-existing membrane protein pool. Consistent with this rationale, MET-targeted VNP simultaneously reduced MET mRNA and protein levels and exhibited more rapid and sustained target suppression than either the siRNA- or DbLYTAC-based modality alone. The therapeutic rationale becomes distinct when different disease-driving proteins are targeted. In the case of MET and EGFR, the objective is not simply to duplicate protein degradation but to block compensatory receptor signaling: suppression of MET alone can promote EGFR-dependent bypass signaling, whereas concurrent MET and EGFR inhibition can more effectively restrain downstream MAPK/ERK signaling. These findings provide experimental support for a context-dependent “1 + 1 > 2” rationale, in which the first layer combines complementary control of mRNA production and protein clearance [76], while the second layer simultaneously disrupts compensatory signaling between distinct oncogenic receptors [77].

### 2.3. Aptamer–ATTEC

**Figure 4:**
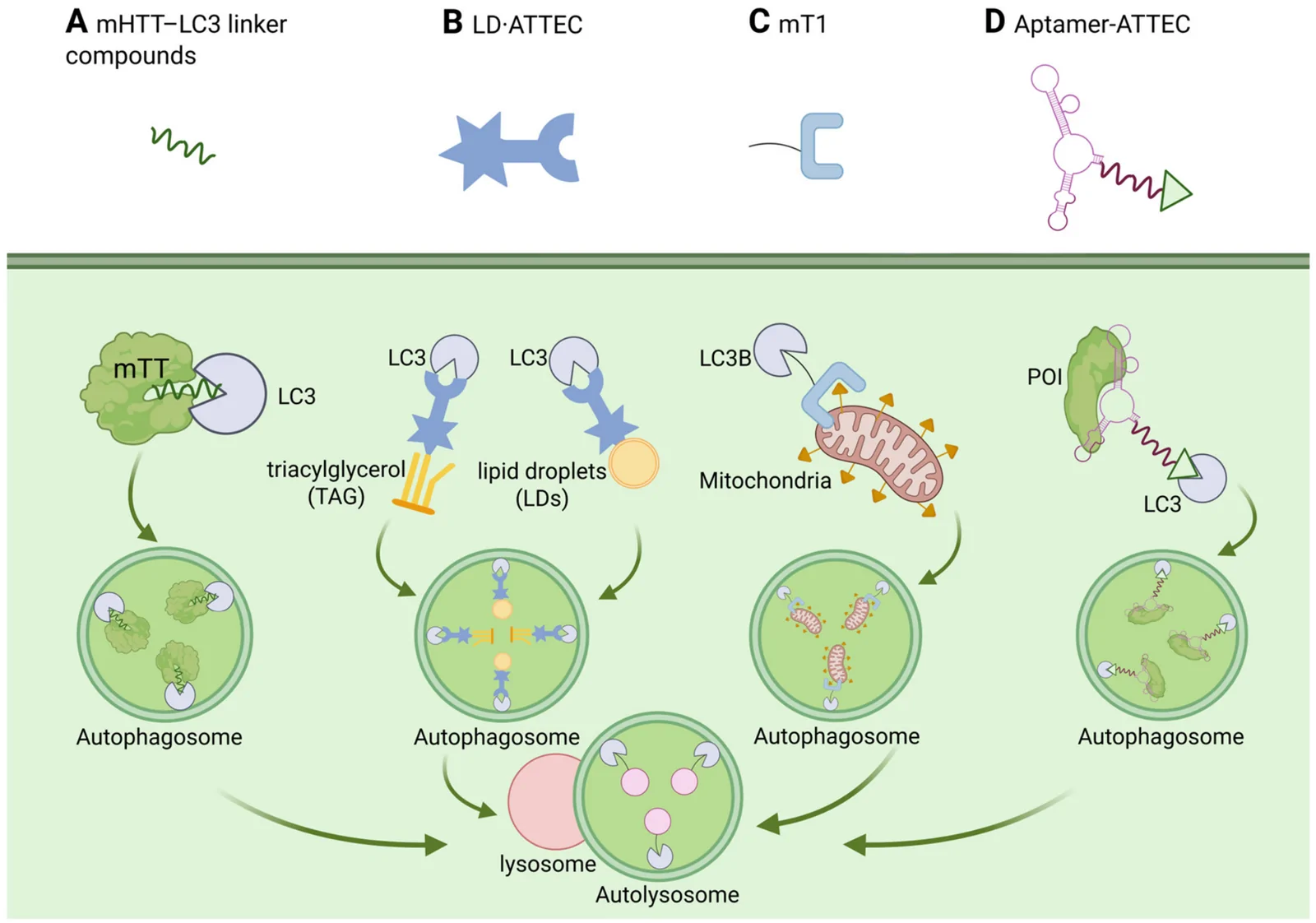
Schematic of aptamer-based ATTECs for targeted degradation via autophagy. Arrows indicate the proposed mechanistic sequence, including formation of degrader–target–effector complexes, target sequestration into autophagosomes, and subsequent autolysosomal degradation. (**A**) mHTT-LC3 linker compounds bind simultaneously to LC3 and to mutant huntingtin protein (mHTT) containing an expanded polyglutamine (polyQ) tract, thereby selectively targeting mHTT for degradation. (**B**) LD·ATTECs mediate lipid droplet (LD) degradation by interacting hydrophobically with neutral lipids (e.g., triacylglycerols, TAG) and with LC3, forming a ternary LD/TAG–LD·ATTEC–LC3 complex that promotes autophagic engulfment and lysosomal breakdown of lipids. (**C**) mT1 simultaneously targets the mitochondrial outer membrane protein TSPO and the autophagosome protein LC3B, thereby tethering mitochondria to autophagosomes and facilitating their degradation via the autophagy-lysosomal pathway. (**D**) General design of an aptamer-ATTEC for targeted protein degradation. The construct comprises an LC3-binding compound, a flexible linker, and a protein-specific aptamer, enabling selective recruitment of the protein of interest (POI) to autophagosomes for lysosomal degradation.

ATTECs represent a distinct class of targeted degradation technologies that utilize the autophagy–lysosome pathway. First conceptualized in 2019 by Lu, this approach was initially demonstrated using the polyQ-binding compound polyQ-ATTEC, which could simultaneously engage the mutant huntingtin protein and the key autophagosome protein LC3 [78]. The core mechanistic principle involved a bifunctional “small-molecule glue” that physically tethered the target substrate, such as disease-causing proteins or lipid droplets, directly to LC3. As LC3 decorates the formation of autophagosomes, this tethering ensures the specific encapsulation of cargo into the vesicle, which subsequently fuses with a lysosome for complete degradation (Figure 4A). The scope of using ATTECs significantly broadened in 2021 when Lu reported a series of LD-ATTEC molecules (C1–C4). These were synthesized by linking known LC3 ligands, such as GW5074 (GW) and 5,7-Dihydroxy-4-phenylcoumarin (DP), to lipid droplet-binding dyes, such as Sudan III/IV, via chemical linkers [79]. These compounds successfully enabled the autophagic clearance of lipid droplets, marking an important expansion of the technology from proteins to non-proteinaceous biomolecules (Figure 4B). A major advancement followed in 2023, with the development of an ATTEC molecule (mT1) designed to target damaged mitochondria. By conjugating the LC3 ligand GW to a ligand for the mitochondrial outer membrane protein TSPO, Lu et al. created a compound that could selectively tag and eliminate impaired mitochondria via autophagy [80]. The ability of ATTECs to degrade organelles facilitates the creation of novel therapeutic strategies addressing multiple mitochondrial disorders (Figure 4C). ATTECs have dramatically expanded the horizon of targeted degradation. Although foundational studies have focused on pathogenic proteins, the modular designs of ATTECs render them highly capable of targeting diverse substrates, including protein aggregates, nucleic acids, other organelles such as peroxisomes and ribosomes, and intracellular pathogens. This versatility establishes ATTECs as pioneering platforms with the capacity to open new research and therapeutic pathways [81].

In contrast, aptamer-based ATTECs are considerably less developed than their conventional small-molecule counterparts. Whereas classical ATTECs rely on small-molecule ligands to engage the substrate, aptamer-ATTECs employ an aptamer as the recognition module, offering a route to targets that are difficult to address with small molecules. Nevertheless, experimental validation of this concept remains scarce. The first clear proof-of-concept was reported by Liao et al., who developed an aptamer–ATTEC designed to selectively degrade pathological α-synuclein (α-syn) in Parkinson’s disease via the autophagy–lysosome pathway (Figure 4D). In this design, a high-affinity α-syn-specific DNA aptamer was conjugated to DP by a click reaction, generating a bifunctional conjugate called DP-Apt. DP-Apt could selectively bind to α-syn and redirect its monomeric and aggregated forms to autophagosomes for degradation. This targeted clearance significantly reduced intracellular α-syn levels, consequently mitigating its associated cytotoxicity and attenuating neuroinflammatory responses [82]. These findings demonstrated that an aptamer can directly serve as the target-recognition moiety of an ATTEC, providing initial proof-of-concept for aptamer-ATTECs. Aptamer-ATTEC represents an emerging extension of the ATTEC field, not a mature degradation platform. Recently, the concept was further applied to the exogenous protein toxin ricin via conjugation of a ricin-binding DNA aptamer to an LC3-recruiting ligand. The resulting aptamer-ATTEC, DP3-D-B0, promoted ternary complex assembly and showed anti-ricin activity across biochemical, cellular, and animal models [83]. Nevertheless, compared with the wealth of data supporting conventional small-molecule ATTECs, the literature on aptamer-ATTECs is still limited. Thus, aptamer-ATTEC should currently be considered a promising but still emerging strategy, with its broader applicability, intracellular delivery, pharmacokinetics, target scope, and therapeutic efficacy requiring further validation.

## 3. Conclusions and Perspectives

Aptamers exhibit high specificity and programmability, offering distinct advantages in molecular recognition, targeted delivery, and therapeutic applications. The ongoing refinement of the SELEX technology, combined with its synergistic integration with computational biology and artificial intelligence, has markedly improved the efficiency of aptamer development and broadened the target repertoire, thereby providing a robust foundation for its use in precision medicine. In parallel, heterobifunctional targeted degraders, such as PROTACs, LYTACs, and ATTECs, exploit endogenous intracellular protein degradation machinery to effectively engage traditionally undruggable targets, substantially expanding the scope of actionable therapeutic interventions.

The intersection of aptamers and heterobifunctional targeted degraders has enabled the emergence of novel degradation strategies, including the APC, aptamer–LYTACs, and aptamer–ATTECs [43]. Importantly, these platforms should not be regarded as a single mechanistic class. Aptamers may function as targeting and delivery modules for conventional PROTACs, as direct POI-binding ligands within a degrader, or as recognition elements that recruit lysosomal or autophagic degradation pathways. This mechanistic diversity substantially expands the potential application of aptamer-based degraders while also creating distinct pharmacological and translational requirements for each platform. They offer substantial benefits in terms of improving tumor specificity, minimizing systemic toxicity, and counteracting drug resistance. Given their promising translational potential, the scope of application of these strategies is rapidly expanding from oncology to diverse therapeutic areas, including neurodegenerative diseases and viral infections. Representative examples of aptamer-based degraders, their molecular targets, degradation mechanisms, disease models, experimental evidence, major outcomes, and current limitations are summarized in Table 1. 

Although the studies summarized in Table 1 demonstrate considerable versatility across intracellular, membrane-associated, and extracellular targets, and recent reviews highlight the promise of aptamer-based heterobifunctional degraders, the current evidence remains predominantly at the proof-of-concept or preclinical stage, with substantial challenges in delivery, stability, pharmacokinetics, and clinical translation. Importantly, the clinical adoption of aptamer therapeutics themselves has been relatively slow. To date, only two aptamer-based therapeutics have received FDA approval: pegaptanib [84], an anti-VEGF RNA aptamer approved in 2004 for neovascular age-related macular degeneration, and avacincaptad pegol [85], a PEGylated RNA aptamer targeting complement C5 that was approved in 2023 for geographic atrophy secondary to age-related macular degeneration. Notably, no aptamer–PROTAC, aptamer–LYTAC, or aptamer–ATTEC has yet received regulatory approval, underscoring the substantial translational gap between the conceptual versatility of these platforms and clinically validated therapeutic products. Several factors contribute to this gap.

Serum instability is a major concern because unmodified DNA or RNA aptamers can undergo nuclease-mediated degradation and rapid renal clearance, thereby limiting systemic exposure and sustained target engagement; chemical modification, terminal protection, backbone/sugar engineering, and multivalent or structurally stabilized architectures are therefore important strategies for improving in vivo persistence [86,87,88]. Although aptamers are generally less immunogenic than protein therapeutics, immunological responses cannot be assumed to be negligible, particularly when chemical modifications, conjugated polymers, nanoparticles, or repeated systemic administration are introduced, making formulation- and construct-specific immunogenicity assessment essential [86,89]. Manufacturing is another important consideration: although sequence-defined chemical synthesis can provide low batch-to-batch variability and potentially lower production costs than antibody manufacturing, the addition of long linkers, modified nucleotides, multivalent scaffolds, targeting ligands, or nanoparticle components can substantially increase process complexity, purification requirements, and quality-control demands [90]. High target affinity does not necessarily translate into productive cellular uptake, endosomal escape, cytosolic access, or appropriate subcellular trafficking, and these requirements become even more stringent when the aptamer is incorporated into a multifunctional degrader construct. Importantly, receptor-mediated uptake should not be equated with productive intracellular delivery. Following endocytosis, aptamer-based constructs may undergo receptor recycling, remain sequestered within endosomal compartments, or traffic toward lysosomes [91]. For aptamer–PROTACs designed to degrade cytosolic or nuclear proteins, productive activity therefore requires not only cellular internalization but also the release of an intact degrader into the cytosol. Existing aptamer–PROTAC studies have generally demonstrated cellular internalization and subsequent target degradation without quantitatively determining the fraction of intact degrader that escapes from endosomes. Thus, the quantitative relationship between uptake, endosomal escape, intracellular degrader availability, and target degradation remains insufficiently characterized and represents an important barrier to rational optimization of aptamer–PROTACs. In contrast, cytosolic escape is not necessarily required for aptamer–LYTACs, for which endosomal trafficking toward lysosomes constitutes the intended intracellular route for degradation of extracellular or membrane-associated targets. For aptamer-based degraders, druggability should therefore be evaluated at the level of the complete construct rather than the aptamer alone.

A further translational challenge is the quantitative control of degradation kinetics, because the pharmacology of targeted degraders is governed not simply by plasma drug concentration but by the dynamic relationship among target engagement, ternary-complex formation, ubiquitination or lysosomal trafficking, protein degradation, and target resynthesis [92,93]. Consequently, future preclinical development should establish integrated PK–PD–degradation models that define parameters such as exposure–degradation relationships, degradation rate, maximal target depletion, recovery kinetics after drug withdrawal, and the duration of pharmacodynamic effects [92]. For aptamer-based systems in particular, these parameters will need to be interpreted together with aptamer–target association and dissociation kinetics, because rapid systemic clearance or loss of target engagement may decouple plasma exposure from the duration of target degradation [87]. Such quantitative characterization will be critical for determining whether sustained target suppression can be achieved without excessive systemic exposure and for establishing rational dosing intervals and therapeutic windows [94].

In parallel, computational biology and artificial intelligence are expected to shift aptamer-degrader development from empirical screening toward structure- and mechanism-guided optimization. For aptamer discovery, AI models can integrate sequence composition, predicted secondary or tertiary structures, and SELEX enrichment information to rank candidate sequences according to their predicted target-binding propensity; for example, DeepAptamer combines convolutional and bidirectional recurrent neural-network architectures with sequence and structural features to predict aptamer binding affinity and identify nucleotides that contribute to target recognition, with experimental validation supporting its predictive utility [95]. Structure-based computational approaches can further combine aptamer folding prediction, molecular docking, and molecular-dynamics simulations to generate and evaluate plausible aptamer–target binding modes, thereby helping prioritize candidates before experimental validation [96]. For the degrader itself, AI can be used to model the spatial relationship between the aptamer/target module and the degradation-recruiting module and to explore linker length, flexibility, and conformational constraints, because these parameters can determine whether a productive ternary or higher-order complex can form. A concrete example is AIMLinker, a deep-learning framework that uses the spatial information of two molecular fragments to generate candidate linkers and subsequently evaluates the resulting structures using docking, molecular dynamics, and free-energy calculations [97]. Nevertheless, these AI-guided approaches should currently be regarded as prioritization and design tools rather than substitutes for experimental validation, because limited high-quality aptamer–target and degrader datasets, conformational heterogeneity, incomplete representation of dynamic ternary complexes, and model generalizability remain important limitations.

Taken together, the future development of aptamer-based heterobifunctional degraders will require integration of molecular recognition, chemical stabilization, delivery engineering, quantitative degradation pharmacology, and AI-assisted structural optimization into a unified developability framework. Rather than optimizing affinity alone, next-generation designs should seek a balanced profile encompassing target selectivity, serum persistence, productive degradation kinetics, tissue exposure, controllable pharmacodynamics, and scalable manufacturing. Such an integrated strategy may facilitate the transition of aptamer-based degraders from proof-of-concept platforms toward clinically actionable precision therapeutics.

## Figures and Tables

**Table 1:** Representative aptamer-based TPD platforms and their current preclinical status.

Degrader/ Platform	Aptamer	Molecular Target	Degradation Machinery/ Receptor Recruited	Disease Model	Evidence	Main Result	Major Limitation(s)
APC	AS1411	BET proteins, particularly BRD4	Conventional small-molecule PROTAC; E3 ligase-dependent UPS	Breast cancer cell; MCF-7 xenograft	In vitro + in vivo	Improved tumor-selective delivery, BET degradation, antitumor efficacy, and reduced toxicity versus unconjugated PROTAC	Aptamer functions mainly as a delivery/targeting module, rather than the POI-binding moiety of the degrader; intracellular release of the conventional PROTAC is required [46].
AS-2F-NP	AS1411-	NAMPT	Conventional NAMPT PROTAC; UPS	Breast cancer cell; MDA-MB-231 xenograft	In vitro + in vivo	Enhanced tumor targeting, cellular uptake, NAMPT degradation, antitumor efficacy and reduced systemic toxicity; enables co-delivery of Fdu and PROTAC	Complex multifunctional architecture; demonstrated mainly in breast cancer models; long-term safety, immune responses and optimal linker design remain unresolved [47].
opto-dNCL#T1	AS1411	NCL	CRBN; UPS	Breast cancer cell; MCF-7/MDA-MB-231 models	In vitro + in vivo for dNCL#T1; in vitro for opto-dNCL#T1	Efficient NCL degradation; opto-dNCL#T1 enables UVA-controlled spatial and temporal activation	Dependence on UVA irradiation limits direct clinical applicability; pharmacokinetics and systemic delivery remain unresolved [48].
dp53m-RA	p53m-RA RNA aptamer	Mutant p53-R175H	CRBN; UPS	p53-R175H-expressing cancer cells	In vitro	Selective degradation of p53-R175H while sparing WT p53 and other p53 mutants; inhibited proliferation and migration	Relatively high degradation concentration (DC50 ≈ 1 µM); no in vivo therapeutic efficacy was established in the original study; nucleic-acid stability and delivery remain concerns [51].
dp53m	p53m-DA DNA aptamer	Mutant p53-R175H	CRBN; UPS	p53-R175H-driven cancer; Detroit 562 cell xenograft model	In vitro + in vivo	Selective mutant-p53 degradation, inhibition of tumor growth, and enhanced cisplatin sensitivity	Still preclinical; translational liabilities of nucleic-acid degraders, including cellular uptake, in vivo stability and PK remain incompletely resolved [52].
circPA1-ProMyc	MA9C1 for c-Myc recognition; PA1 for PD-L1 recognition/targeted delivery	c-Myc and PD-L1	CRBN; UPS	HCT116 colorectal cancer cells; HCT116 xenograft tumors in BALB/c nude mice	In vitro + in vivo	Artificial cyclization improved serum stability, while PA1 promoted internalization into PD-L1-positive cells. circPA1-ProMyc simultaneously decreased c-Myc and PD-L1; its antitumor activity was primarily attributed to c-Myc degradation. Peritumoral administration significantly suppressed xenograft tumor growth without significant body-weight loss.	Still preclinical; unresolved issues include the complex dual-aptamer design, peri-tumoral rather than systemic delivery, and validation in immunocompetent models [55].
AHPC-cb-Ap	SL1, a c-Met-targeting aptamer; Mj5c, a PD-L1-targeting aptamer	c-Met and EGFR; PD-L1	UPS, mediated by two AHPC E3-ligase-recruiting ligands	Erlotinib-resistant NCI-H1975 NSCLC cells and NCI-H1975 tumor-bearing mice	In vitro + in vivo	Simultaneous c-Met and EGFR degradation, restoration of erlotinib sensitivity in resistant cells/tumors, and preclinical efficacy comparable to osimertinib.	The construct is structurally complex and incorporates two aptamers and two E3-recruiting ligands. Current evidence is focused on EGFR-TKI-resistant NSCLC, and systemic pharmacokinetics, biodistribution, long-term safety, and broader therapeutic applicability remain to be established [57].
dFUS-E9C (Oligo-PROTAC)	FUS-RNA#E9	Disease-associated FUS and cytoplasmic FUS aggregates	CRBN; UPS	PC12, SH-SY5Y, Neuro-2a and HEK-293T cellular models; BALB/c mice for brain delivery/degradation	In vitro + in vivo	dFUS-E9C showed high-affinity FUS binding (Kd ≈ 60.54 nM) and CRBN/proteasome-dependent degradation; preferentially removed cytoplasmic FUS aggregates and rescued FUS-induced mitochondrial dysfunction. The DNA-nanoflower formulation FRONTAC^FUS^ crossed the BBB after intravenous administration and reduced brain FUS by >50–70%, with effects persisting for up to 14 days.	dFUS-E9C itself is an Oligo-PROTAC rather than a classical SELEX-derived aptamer-PROTAC. Systemic brain delivery required additional phosphorothioate modification and a TfR-targeting DNA-nanoflower carrier, substantially increasing construct complexity. Therapeutic efficacy in a bona fide FUS-ALS/FTD disease model remains to be established [60].
C-PROTAC	Z3	ZBP1	VHL; UPS	H1N1-infected cellular models; influenza A virus-infected mice	In vitro + in vivo	Covalent aptamer–ZBP1 engagement produced substantially stronger degradation than the non-covalent counterpart; suppressed ZBP1-associated inflammatory signaling and improved cell viability. In infected mice, treatment increased survival to approximately 80% versus 20% in untreated infected controls and reduced pulmonary inflammation/edema	Covalent target engagement may reduce degrader recyclability and raises the need for careful assessment of irreversible off-target interactions and long-term safety. In addition, the in vivo formulation relied on liposomal delivery, so the therapeutic contribution of the degrader itself versus the delivery system requires further optimization [65].
Apt-LYTAC	PDGF- or PTK7-binding aptamers	PDGF; PTK7	Tri-GalNAc–ASGPR; lysosome	HepG2 and related cellular models	In vitro	Efficient lysosomal degradation of extracellular PDGF and membrane PTK7	ASGPR-dependent activity restricts effective targeting largely to ASGPR-positive cells; significant hook effect observed in dose–response curves; lack of any in vivo efficacy or toxicity studies [72].
DBCO-chimera	PD-L1-binding aptamer + CI-M6PR binding aptamer	PD-L1	CI-M6PR; lysosome	B16F10, CT26 and 4T1 tumor models	In vitro + in vivo	Covalent target engagement improved PD-L1 degradation and antitumor activity and induced immunogenic apoptosis	Covalent chemistry increases design complexity; the study remains a proof-of-concept preclinical platform and broader target/receptor generalizability remains to be established [73].
VED-LYTAC	VEGF aptamer + M6PR aptamer	VEGF	M6PR; lysosome	Neovascular ocular disease; mouse retinal neovascularization models	In vitro + ex vivo + in vivo	Reduced VEGF activity, pathological angiogenesis and vascular leakage after intravitreal administration	Requires local intravitreal administration; systemic applicability, long-term ocular safety and pharmacokinetics remain to be established [74].
PBAC	Sgc8 + CI-M6PR binding aptamer	PTK7	IGFIIR; lysosome	Colorectal tumor xenograft model; HCT 116 tumor-bearing mouse model	In vitro + in vivo	NIR-triggered degradation of PTK7 and strong suppression of colorectal tumor growth	Requires external NIR irradiation; multifunctional design increases complexity and may constrain clinical translation [75].
DbLYTAC	MET- or EGFR-targeting aptamer + M6PR-targeting aptamer	MET/EGFR	M6PR; lysosome; combined with siRNA-mediated gene silencing	MHCC97h liver cancer cell; MHCC97h tumor-bearing mice	In vitro + in vivo	Combined membrane-protein degradation and gene silencing; enhanced antitumor efficacy and modular dual-targeting capability	Complex preparation processes, high immunogenicity of the protein shell, inadequate stability, and sensitivity to environmental conditions such as temperature and pH [76].
DP-Apt	α-synuclein aptamer	α-Synuclein	LC3; autophagy–lysosome pathway	Parkinson’s disease cell models based on overexpression of α-synuclein (wild-type or A53T mutant) in HEK293T, HT22, MCF-7 and U-87 cells	In vitro	Degraded monomeric and aggregated α-synuclein and reduced associated cytotoxicity/neuroinflammatory responses	Early proof-of-concept; limited target scope and lack of demonstrated in vivo therapeutic efficacy in the original study [82].
DP3-D-B0	Ricin-binding DNA aptamer	Ricin	LC3; autophagy–lysosome pathway	Ricin intoxi-cation mouse models	Molecular + cellular + animal	Promoted LC3–ATTEC–ricin complex formation and lysosomal degradation; showed anti-ricin efficacy in vivo	Very recent proof-of-concept; target is an exogenous toxin rather than a conventional endogenous therapeutic POI, so generalizability to disease-associated proteins remains uncertain [83].

## Data Availability

No new data were created or analyzed in this study. Data sharing is not applicable to this article.

## References

[1] Tuerk C., Gold L. (1990). Systematic evolution of ligands by exponential enrichment: RNA ligands to bacteriophage t4 DNA polymerase. Science.

[2] Li W., Zhou L., Zheng M., Fang J. (2018). Selection of metastatic breast cancer cell-specific aptamers for the capture of CTCs with a metastatic phenotype by cell-selex. Mol. Ther. Nucleic Acids.

[3] Mayer G. (2009). The chemical biology of aptamers. Angew. Chem. Int. Ed..

[4] Zhang L., Wang M., Zhu Z., Ding C., Chen S., Wu H., Yang Y., Che F., Li Q., Li H. (2021). A novel ph-sensitive multifunctional DNA nanomedicine: An enhanced and harmless gd2 aptamer-mediated strategy for guiding neuroblastoma antitumor therapy. Int. J. Nanomed..

[5] Li L., Xie S., Zhou J., Ran J. (2025). Utilizing aptamers in targeted protein degradation strategies for disease therapy. J. Pathol..

[6] Wen C., Wang Y., Wang Y. (2025). Aptamers in drug delivery development. Mater. Today.

[7] Coppola G., Cennamo F., Ciccone G., Ibba M.L., Ruscio A.D., Vito A.D., Esposito C.L., Catuogno S. (2025). Aptamer-based applications in delivering cancer gene therapies and beyond: State of the art and the missing links to clinical translation. Adv. Drug Deliv. Rev..

[8] Levy-Nissenbaum E., Radovic-Moreno A.F., Wang A.Z., Langer R., Farokhzad O.C. (2008). Nanotechnology and aptamers: Applications in drug delivery. Trends Biotechnol..

[9] Abune L., Davis B., Wang Y. (2021). Aptamer-functionalized hydrogels: An emerging class of biomaterials for protein delivery, cell capture, regenerative medicine, and molecular biosensing. Wiley Interdiscip. Rev. Nanomed. Nanobiotechnol..

[10] Zhou J., Battig M.R., Wang Y. (2010). Aptamer-based molecular recognition for biosensor development. Anal. Bioanal. Chem..

[11] Xu W., Liu Q., Sun N., Gao T., Pei R. (2026). Protein-targeted aptamers in liquid biopsy: From efficient screening to precise cancer diagnosis. Analyst.

[12] Liu C., Zhao J., Tian F., Cai L., Zhang W., Feng Q., Chang J., Wan F., Yang Y., Dai B. (2019). Low-cost thermophoretic profiling of extracellular-vesicle surface proteins for the early detection and classification of cancers. Nat. Biomed. Eng..

[13] Cao J., Zhang F., Xiong W. (2023). Discovery of aptamers and the acceleration of the development of targeting research in ophthalmology. Int. J. Nanomed..

[14] Sun M., Liu S., Wei X., Wan S., Huang M., Song T., Lu Y., Weng X., Lin Z., Chen H. (2021). Aptamer blocking strategy inhibits SARS-COV-2 virus infection. Angew. Chem. Int. Ed..

[15] Liang Z., Peng Y., Chen Y., Long L., Luo H., Chen Y., Liang Y., Tian Y., Li S., Shi Y. (2019). The bace1-specific DNA aptamer a1 rescues amyloid-β pathology and behavioral deficits in a mouse model of alzheimer’s disease. Nucleic Acid Ther..

[16] Xiang J., Zhang W., Cai X., Cai M., Yu Z., Yang F., Zhu W., Li X., Wu T., Zhang J. (2019). DNA aptamers targeting bace1 reduce amyloid levels and rescue neuronal deficiency in cultured cells. Mol. Ther. Nucleic Acids.

[17] Kim J., Na H., Choi S., Oh E.J., Lee H., Ryu S.H., Yunn N., Cho Y. (2025). Structural mechanism of insulin receptor activation by a dimeric aptamer agonist. Exp. Mol. Med..

[18] Verma Y., Khan I., Dongsar T.T., Alsayari A., Wahab S., Sahebkar A., Kesharwani P. (2025). As1411 aptamer based nanomaterials: A novel approach for breast cancer therapy. Int. J. Biol. Macromol..

[19] Bekes M., Langley D.R., Crews C.M. (2022). Protac targeted protein degraders: The past is prologue. Nat. Rev. Drug Discov..

[20] Henley M.J., Koehler A.N. (2021). Advances in targeting ‘undruggable’ transcription factors with small molecules. Nat. Rev. Drug Discov..

[21] Moore A.R., Rosenberg S.C., Mccormick F., Malek S. (2020). Ras-targeted therapies: Is the undruggable drugged?. Nat. Rev. Drug Discov..

[22] Zhong G., Chang X., Xie W., Zhou X. (2024). Targeted protein degradation: Advances in drug discovery and clinical practice. Signal Transduct. Target. Ther..

[23] Zhao L., Zhao J., Zhong K., Tong A., Jia D. (2022). Targeted protein degradation: Mechanisms, strategies and application. Signal Transduct. Target. Ther..

[24] Lu D., Yu X., Lin H., Cheng R., Monroy E.Y., Qi X., Wang M.C., Wang J. (2022). Applications of covalent chemistry in targeted protein degradation. Chem. Soc. Rev..

[25] Li P., Jia C., Fan Z., Hu X., Zhang W., Liu K., Sun S., Guo H., Yang N., Zhu M. (2023). Discovery of novel exceptionally potent and orally active c-met protacs for the treatment of tumors with met alterations. Acta Pharm. Sin. B.

[26] Liu J., Farmer J.D.J., Lane W.S., Friedman J., Weissman I., Schreiber S.L. (1991). Calcineurin is a common target of cyclophilin-cyclosporin a and fkbp-fk506 complexes. Cell.

[27] Sakamoto K.M., Kim K.B., Kumagai A., Mercurio F., Crews C.M., Deshaies R.J. (2001). Protacs: Chimeric molecules that target proteins to the skp1-cullin-f box complex for ubiquitination and degradation. Proc. Natl. Acad. Sci. USA.

[28] Banik S.M., Pedram K., Wisnovsky S., Ahn G., Riley N.M., Bertozzi C.R. (2020). Lysosome-targeting chimaeras for degradation of extracellular proteins. Nature.

[29] Ji C.H., Kim H.Y., Lee M.J., Heo A.J., Park D.Y., Lim S., Shin S., Ganipisetti S., Yang W.S., Jung C.A. (2022). The autotac chemical biology platform for targeted protein degradation via the autophagy-lysosome system. Nat. Commun..

[30] Campone M., De Laurentiis M., Jhaveri K., Hu X., Ladoire S., Patsouris A., Zamagni C., Cui J., Cazzaniga M., Cil T. (2025). Vepdegestrant, a protac estrogen receptor degrader, in advanced breast cancer. N. Engl. J. Med..

[31] Wang W., Zhou Q., Jiang T., Li S., Ye J., Zheng J., Wang X., Liu Y., Deng M., Ke D. (2021). A novel small-molecule protac selectively promotes tau clearance to improve cognitive functions in alzheimer-like models. Theranostics.

[32] Song Y., Zhou B., Long J., Peng Y. (2025). Targeted protein degradation in autoimmune diseases: From mechanisms to therapeutic breakthroughs. J. Autoimmun..

[33] Peng X., Hu Z., Zeng L., Zhang M., Xu C., Lu B., Tao C., Chen W., Hou W., Cheng K. (2024). Overview of epigenetic degraders based on protac, molecular glue, and hydrophobic tagging technologies. Acta Pharm. Sin. B.

[34] Chen Y., Tandon I., Heelan W., Wang Y., Tang W., Hu Q. (2022). Proteolysis-targeting chimera (protac) delivery system: Advancing protein degraders towards clinical translation. Chem. Soc. Rev..

[35] Moreau K., Coen M., Zhang A.X., Pachl F., Castaldi M.P., Dahl G., Boyd H., Scott C., Newham P. (2020). Proteolysis-targeting chimeras in drug development: A safety perspective. Br. J. Pharmacol..

[36] Ding Y., Liu J. (2024). Kinetic ITC of DNA aptamers binding for small molecules and implications for binding assays and biosensors. ChemBioChem A Eur. J. Chem. Biol..

[37] Mahmoudian F., Ahmari A., Shabani S., Sadeghi B., Fahimirad S., Fattahi F. (2024). Aptamers as an approach to targeted cancer therapy. Cancer Cell Int..

[38] Thiel W.H., Bair T., Peek A.S., Liu X., Dassie J., Stockdale K.R., Behlke M.A., Miller F.J.J., Giangrande P.H. (2012). Rapid identification of cell-specific, internalizing RNA aptamers with bioinformatics analyses of a cell-based aptamer selection. PLoS ONE.

[39] Gissberg O., Zaghloul E.M., Lundin K.E., Nguyen C., Landras-Guetta C., Wengel J., Zain R., Smith C.I.E. (2016). Delivery, effect on cell viability, and plasticity of modified aptamer constructs. Nucleic Acid Ther..

[40] Tanaka K., Okuda T., Kasahara Y., Obika S. (2020). Base-modified aptamers obtained by cell-internalization selex facilitate cellular uptake of an antisense oligonucleotide. Mol. Ther. Nucleic Acids.

[41] Xiao Z., Levy-Nissenbaum E., Alexis F., Lupták A., Teply B.A., Chan J.M., Shi J., Digga E., Cheng J., Langer R. (2012). Engineering of targeted nanoparticles for cancer therapy using internalizing aptamers isolated by cell-uptake selection. ACS Nano.

[42] Luo H., Tian Y., Abdullah R., Zhang B., Ma Y., Zhang G. (2025). Advancing design strategy of protacs for cancer therapy. MedComm.

[43] Liu Y., Qian X., Ran C., Li L., Fu T., Su D., Xie S., Tan W. (2023). Aptamer-based targeted protein degradation. ACS Nano.

[44] Yang Z., Pang Q., Zhou J., Xuan C., Xie S. (2023). Leveraging aptamers for targeted protein degradation. Trends Pharmacol. Sci..

[45] Li K., Crews C.M. (2022). Protacs: Past, present and future. Chem. Soc. Rev..

[46] He S., Gao F., Ma J., Ma H., Dong G., Sheng C. (2021). Aptamer-protac conjugates (APCs) for tumor-specific targeting in breast cancer. Angew. Chem. Int. Ed..

[47] He S., Fang Y., Zhu Y., Ma Z., Dong G., Sheng C. (2024). Drugtamer-protac conjugation strategy for targeted protac delivery and synergistic antitumor therapy. Adv. Sci..

[48] Chen M., Zhou P., Kong Y., Li J., Li Y., Zhang Y., Ran J., Zhou J., Chen Y., Xie S. (2023). Inducible degradation of oncogenic nucleolin using an aptamer-based protac. J. Med. Chem..

[49] Chen S., Gao R., Yao C., Kobayashi M., Liu S.Z., Yoder M.C., Broxmeyer H., Kapur R., Boswell H.S., Mayo L.D. (2018). Genotoxic stresses promote clonal expansion of hematopoietic stem cells expressing mutant p53. Leukemia.

[50] Chiang Y., Chien Y., Lin Y., Wu H., Lee D., Yu Y. (2021). The function of the mutant p53-r175h in cancer. Cancers.

[51] Kong L., Meng F., Wu S., Zhou P., Ge R., Liu M., Zhang L., Zhou J., Zhong D., Xie S. (2023). Selective degradation of the p53-r175h oncogenic hotspot mutant by an RNA aptamer-based protac. Clin. Transl. Med..

[52] Kong L., Meng F., Zhou P., Ge R., Geng X., Yang Z., Li G., Zhang L., Wang J., Ma J. (2024). An engineered DNA aptamer-based protac for precise therapy of p53-r175h hotspot mutant-driven cancer. Sci. Bull..

[53] Madden S.K., de Araujo A.D., Gerhardt M., Fairlie D.P., Mason J.M. (2021). Taking the myc out of cancer: Toward therapeutic strategies to directly inhibit c-myc. Mol. Cancer.

[54] Casacuberta-Serra S., González-Larreategui Í.I., Capitán-Leo D., Soucek L. (2024). Myc and kras cooperation: From historical challenges to therapeutic opportunities in cancer. Signal Transduct. Target. Ther..

[55] Wang Y., Yang G., Zhang X., Bai R., Yuan D., Gao D., He Q., Yuan Y., Zhang X., Kou J. (2024). Antitumor effect of anti-c-myc aptamer-based protac for degradation of the c-myc protein. Adv. Sci..

[56] Ichihara E., Westover D., Meador C.B., Yan Y., Bauer J.A., Lu P., Ye F., Kulick A., de Stanchina E., Mcewen R. (2017). SFK/FAK signaling attenuates osimertinib efficacy in both drug-sensitive and drug-resistant models of EGFR-mutant lung cancer. Cancer Res..

[57] Wu S., Zhou X., Ai L., Shang Y., Yan Y., Zhao Z., Tan W. (2025). A cyclized bivalent aptamer-based protein degrader targeting receptor tyrosine kinases overcomes resistance to inhibitors in lung cancer. ACS Nano.

[58] Mackenzie I.R., Rademakers R., Neumann M. (2010). Tdp-43 and fus in amyotrophic lateral sclerosis and frontotemporal dementia. Lancet Neurol..

[59] Nolan M., Talbot K., Ansorge O. (2016). Pathogenesis of fus-associated als and ftd: Insights from rodent models. Acta Neuropathol. Commun..

[60] Ge R., Chen M., Wu S., Huang S., Zhou P., Cao M., Zhang F., Zang J., Zhu Y., Li J. (2025). DNA nanoflower oligo-protac for targeted degradation of fus to treat neurodegenerative diseases. Nat. Commun..

[61] Newton A.H., Cardani A., Braciale T.J. (2016). The host immune response in respiratory virus infection: Balancing virus clearance and immunopathology. Semin. Immunopathol..

[62] Zhang T., Yin C., Boyd D.F., Quarato G., Ingram J.P., Shubina M., Ragan K.B., Ishizuka T., Crawford J.C., Tummers B. (2020). Influenza virus z-RNAs induce zbp1-mediated necroptosis. Cell.

[63] Upton J.W., Kaiser W.J., Mocarski E.S. (2019). DAI/ZBP1/DLM-1 complexes with rip3 to mediate virus-induced programmed necrosis that is targeted by murine cytomegalovirus vira. Cell Host Microbe.

[64] Maelfait J., Rehwinkel J. (2023). The z-nucleic acid sensor ZBP1 in health and disease. J. Exp. Med..

[65] Huang R., Hu Y., Wang Y., Zhang S., Wang Z., Pang D., Liu S. (2025). Targeted degradation of ZBP1 with covalent protacs for anti-inflammatory treatment of infections. Angew. Chem. Int. Ed..

[66] Kiely-Collins H., Winter G.E., Bernardes G.A.J.L. (2021). The role of reversible and irreversible covalent chemistry in targeted protein degradation. Cell Chem. Biol..

[67] Gabizon R., Shraga A., Gehrtz P., Livnah E., Shorer Y., Gurwicz N., Avram L., Unger T., Aharoni H., Albeck S. (2020). Efficient targeted degradation via reversible and irreversible covalent protacs. J. Am. Chem. Soc..

[68] Tan J., Liang Y., Shao S., Shen Y. (2026). Covalent chemistry in targeted protein degradation. Adv. Drug Deliv. Rev..

[69] Zhang K., Qiu F., Lyu A., Liang C. (2026). Extracellular and membrane-targeted protein degradation (emtpd): Mechanisms, design frameworks, and therapeutic opportunities. Innov. Drug Discov..

[70] Ahn G., Banik S.M., Miller C.L., Riley N.M., Cochran J.R., Bertozzi C.R. (2021). Lytacs that engage the asialoglycoprotein receptor for targeted protein degradation. Nat. Chem. Biol..

[71] Yang Z., Xu J., Yang X., Chen J. (2025). Targeted protein degradation with small molecules for cancer immunotherapy. Asian J. Pharm. Sci..

[72] Wu Y., Lin B., Lu Y., Li L., Deng K., Zhang S., Zhang H., Yang C., Zhu Z. (2023). Aptamer-lytacs for targeted degradation of extracellular and membrane proteins. Angew. Chem. Int. Ed..

[73] Li Y., Liu X., Yu L., Huang X., Wang X., Han D., Yang Y., Liu Z. (2023). Covalent lytac enabled by dna aptamers for immune checkpoint degradation therapy. J. Am. Chem. Soc..

[74] Zhou P., Zhang S., Li L., Zhang R., Guo G., Zhang Y., Wang R., Liu M., Wang Z., Zhao H. (2024). Targeted degradation of vegf with bispecific aptamer-based lytacs ameliorates pathological retinal angiogenesis. Theranostics.

[75] Zhang R., Yang C., Gao X., He Z., Ji D., Tan W. (2025). Phototriggered lytac: Photoactive bispecific aptamer chimera enhances targeted degradation of membrane protein through regulating cell autophagy. J. Am. Chem. Soc..

[76] Dou Q., Wang J., Mao M., Shui L., Hu T.Y., Zhang Y. (2025). Engineered virus-like nanoparticles enable multimodal protein degradation for enhanced tumor therapy. Adv. Mater..

[77] Yang L., Bhattacharya A., Peterson D., Li Y., Liu X., Marangoni E., Robila V., Zhang Y. (2024). Targeted dual degradation of her2 and EGFR obliterates oncogenic signaling, overcomes therapy resistance, and inhibits metastatic lesions in her2-positive breast cancer models. Drug Resist. Updat. Rev. Comment. Antimicrob. Anticancer Chemother..

[78] Li Z., Wang C., Wang Z., Zhu C., Li J., Sha T., Ma L., Gao C., Yang Y., Sun Y. (2019). Allele-selective lowering of mutant htt protein by htt-lc3 linker compounds. Nature.

[79] Fu Y., Chen N., Wang Z., Luo S., Ding Y., Lu B. (2021). Degradation of lipid droplets by chimeric autophagy-tethering compounds. Cell Res..

[80] Tan S., Wang D., Fu Y., Zheng H., Liu Y., Lu B. (2023). Targeted clearance of mitochondria by an autophagy-tethering compound (attec) and its potential therapeutic effects. Sci. Bull..

[81] Ding Y., Fei Y., Lu B. (2020). Emerging new concepts of degrader technologies. Trends Pharmacol. Sci..

[82] Liao X., Qin G., Liu Z., Ren J., Qu X. (2024). Bioorthogonal aptamer-attec conjugates for degradation of alpha-synuclein via autophagy-lysosomal pathway. Small.

[83] Zhang J., Xu Z., Liu S., Wang X., Wang F., Chen Y., Ma X., Li D., Zheng A., Shi W. (2026). An innovative ricin-degrading antidote based on aptamer-autophagy-tethering compound strategy. Int. J. Biol. Macromol..

[84] Ng E.W.M., Shima D.T., Calias P., Cunningham E.T.J., Guyer D.R., Adamis A.P. (2006). Pegaptanib, a targeted anti-vegf aptamer for ocular vascular disease. Nat. Rev. Drug Discov..

[85] Mullard A. (2023). FDA approves second RNA aptamer. Nat. Rev. Drug Discov..

[86] Zhou J., Rossi J. (2017). Aptamers as targeted therapeutics: Current potential and challenges. Nat. Rev. Drug Discov..

[87] Pan Z., Pan Y., Zhang H., Chen Z., Zhang Y., Zhang B., Lu A., Zhang G., Yu S. (2026). Stability modification of therapeutic aptamers: From biostability bottlenecks to nuclease-resistant construct design. RSC Chem. Biol..

[88] Ni S., Yao H., Wang L., Lu J., Jiang F., Lu A., Zhang G. (2017). Chemical modifications of nucleic acid aptamers for therapeutic purposes. Int. J. Mol. Sci..

[89] Ren G., Xu R., Zhao L., Ning L., Zhang H., Sun X., Lu Z., Liu X., Jia F. (2026). Brush-on-brush architecture enables durable and reversible aptamer therapeutics with minimal peg associated immunogenicity. Adv. Mater..

[90] Ni S., Zhuo Z., Pan Y., Yu Y., Li F., Liu J., Wang L., Wu X., Li D., Wan Y. (2021). Recent progress in aptamer discoveries and modifications for therapeutic applications. Acs Appl. Mater. Interfaces.

[91] Juliano R.L. (2018). Intracellular trafficking and endosomal release of oligonucleotides: What we know and what we don’t. Nucleic Acid Ther..

[92] Bartlett D.W., Gilbert A.M. (2022). Translational pk-pd for targeted protein degradation. Chem. Soc. Rev..

[93] Tsai J.M., Nowak R.A.P., Ebert B.L., Fischer E.S. (2024). Targeted protein degradation: From mechanisms to clinic. Nat. Rev. Mol. Cell Biol..

[94] Pike A., Lee E.C.Y., Michaelides I.N., Schade M., Sharma A., Scott J.S., Srivastava A. (2026). Lessons learned in linking protacs from discovery to the clinic. Nat. Rev. Chem..

[95] Yang X., Chan C.H., Yao S., Chu H.Y., Lyu M., Chen Z., Xiao H., Ma Y., Yu S., Li F. (2024). Deepaptamer: Advancing high-affinity aptamer discovery with a hybrid deep learning model. Mol. Ther. Nucleic Acids.

[96] Chen Z., Hu L., Zhang B., Lu A., Wang Y., Yu Y., Zhang G. (2021). Artificial intelligence in aptamer-target binding prediction. Int. J. Mol. Sci..

[97] Kao C., Lin C., Chou C., Lin C. (2023). Fragment linker prediction using the deep encoder-decoder network for protacs drug design. J. Chem. Inf. Model..

